# Guanxinning Tablet Attenuates Coronary Atherosclerosis via Regulating the Gut Microbiota and Their Metabolites in Tibetan Minipigs Induced by a High-Fat Diet

**DOI:** 10.1155/2022/7128230

**Published:** 2022-07-28

**Authors:** Qinqin Yang, Yanyun Xu, Liye Shen, Yongming Pan, Junjie Huang, Quanxin Ma, Chen Yu, Jiaojiao Chen, Yu Chen, Minli Chen

**Affiliations:** ^1^Academy of Chinese Medicine & Institute of Comparative Medicine, Zhejiang Chinese Medical University, Hangzhou, China; ^2^Department of Experimental Animals, Zhejiang Academy of Traditional Chinese Medicine, Hangzhou, China; ^3^School of Pharmaceutical Sciences, Zhejiang Chinese Medical University, Hangzhou, China

## Abstract

Coronary atherosclerosis (CA) is a chronic and evolving inflammatory disease characterized by the build-up of atherosclerotic plaque in the wall of coronary arteries. Guanxinning tablet (GXNT) is a novel Chinese medicine formula, which has been clinically used to treat coronary heart disease for many years. However, the potential mechanism for treating CA remains unclear. Thus, the study was aimed at investigating the therapeutic effect of GXNT on CA and further explore the underlying mechanisms from the perspective of gut microbiota. Following the establishment of a CA model in Tibetan minipigs, GXNT was orally administrated. We simultaneously detected blood lipid levels, observed ventricular function using ultrasound examination, measured platelet aggregation, and checked changes in inflammatory factors, oxidative stress factors, and vascular endothelial injury-related indexes applying ELISA assays. Histopathological changes of coronary artery tissue were subsequently evaluated using Sudan IV staining, HE staining, Oil red “O” staining, and immunohistochemistry assays. Finally, alterations of the gut microbiota and microbial metabolites were detected using metagenomic sequencing and targeted metabolomics, respectively. The results have suggested that GXNT could regulate dyslipidemia, improve heart function, and inhibit the levels of ox-LDL, CRP, TNF-*α*, IL-1*β*, SOD, MDA, vWF, and ET-1, as well as platelet aggregation. Additionally, histopathological findings revealed that GXNT could reduce lipid deposition, alleviate AS lesions, and restrain the expressions of NF-*κ*B, TNF-*α*, and MMP-9. Furthermore, the composition of the gut microbiota was altered. Specifically, GXNT could upregulate the relative abundance of *Prevotellaceae* and *Prevotella* and downregulate the abundance of *Proteobacteria*, *Enterobacteriaceae*, and *Escherichia*. As for microbial metabolites, GXNT could increase fecal propionic acid, butyric acid, and LCA-3S and decrease fecal TMA-related metabolites, CDCA, and serum TMAO. In sum, the results showed that GXNT had a satisfactory anti-CA effect, and the mechanism was closely associated with modulating gut microbiota and related metabolites.

## 1. Introduction

Coronary atherosclerosis (CA) continues to be a leading cause of illness and death worldwide, which refers to a chronic inflammatory pathological change in the wall of coronary arteries [1]. It causes vascular lumen stenosis or thrombosis, resulting in angina pectoris, myocardial infarction, and even sudden death. Atherosclerosis (AS), as the pathological basis of CA, is characterized by progressive lipid deposition, inflammatory cell infiltration, and fibrosis [2]. The occurrence and progression of CA are attributable to genetic and environmental factors, such as dyslipidemia, metabolic syndrome, and air pollution, whereas the mechanisms are still noncompletely understood.

Recently, emerging studies reveal that gut microbes are likely to be one of the important pathogenic factors for CA. Gut microbiota participates in host nutrient absorption and metabolism. It can not only affect glucose and lipid metabolism by directly regulating energy intake in food but also affect the circulating endotoxin level and inflammatory response by indirectly changing intestinal permeability, thereby facilitating CA development [3]. Liu et al. have found that the homeostasis of the gut microbiome in CA patients was disrupted, accompanied by significant changes in the composition of microbes and metabolites [4]. Once the integrity of the gut barrier is broken, the gut microbiota and metabolites are more likely to enter the blood circulation, followed by chronic systemic inflammatory responses and AS formation. Accumulating evidence suggests that the microbiota-dependent metabolite trimethylamine-N-oxide (TMAO) promotes AS formation, and diet-induced inflammation will be alleviated if a TMAO inhibitor is applied [5]. In addition, it is reported that the proportion of *Bacteroidetes* and *Firmicutes* changed in CA patients, as well as gene functions, including lipopolysaccharide biosynthesis, fermentative capacity, and propanoate metabolism [6]. These findings hint that differences in the structure and function of the gut microbiota may affect CA.

Guanxinning tablet (GXNT) is a new preparation of Chinese medicine formula, developed from GuanXinNing injection by changing the route of medication. GXNT has a unique therapeutic effect on cardiovascular diseases, especially coronary heart disease (CHD), which is reported to be a safe and effective treatment for stable angina pectoris patients [7]. GXNT consists of two traditional Chinese medicines, *Salvia miltiorrhiza* Bunge. and *Ligusticum chuanxiong* Hort. in a 1 : 1 ratio, possessing the effects of activating blood circulation, removing stasis, dredging arteries, and nourishing the heart [8]. Our previous studies have found that GXNT contains a variety of phenolic acid active components, and salvianolic acid B in *Salvia miltiorrhiza* Bunge. and ferulic acid in *Ligusticum chuanxiong* Hort. are used as the quality control specification. Despite these active ingredients having low oral bioavailability [9, 10], they can still exert efficacy, so we infer that the mechanism may be related to the gut microbiota. The Tibetan minipig CA model induced by a high-fat (HF) diet is an ideal model for simulating the formation of human AS plaques. We preliminarily found that GXNT could improve HF diet-induced chronic low-grade inflammation and inhibit AS formation in minipigs, suggesting that GXNT has an anti-AS effect [11]. From this, our study observed the efficacy of GXNT on long-term HF diet-induced CA in Tibetan minipigs and explored the potential mechanism by which GXNT treats CA based on the gut microbiota.

## 2. Materials and Methods

### 2.1. Animals

Male Tibetan minipigs, aged 4-5 months and weighing approximately 8-12 kg, were purchased from Dongguan Songshan Lake Pearl Laboratory Animal Science and Technology Co., Ltd. (Dongguan, China, No. 44410500000286). All minipigs were raised in the Laboratory Animal Research Center of Zhejiang Chinese Medical University (Hangzhou, China) under the conditions of temperature: 22 ± 1°C, relative humidity: 40% ~65%, and 12 h/12 h light and dark cycle. They were fed two meals a day and had free access to water. All animal experiment procedures in this study were authorized by the Committee on the Management and Use of Laboratory Animals (IACUC Approval No. 20191021-10) and conducted in line with “3R” principles of animal welfare.

### 2.2. Modeling and Drug Administration

GXNT was provided by Chiatai Qinchunbao Pharmaceutical co., Ltd. (Hangzhou, China). 14 active ingredients were identified, and 7 main active substances of phenolic acids were quantified in GXNT using liquid chromatography-mass spectrometry (LC-MS) [12] (see details in Supplementary Table 1). The drug was mixed into a HF diet and fed twice a day. After adaptive feeding, 18 Tibetan minipigs were selected according to fasting biochemical indicators. Of which, 6 were fed a normal diet and served as the control group. And the other 12 were fed a HF diet (formula: 1.5% cholesterol, 15% shortening, 10% egg yolk powder, 0.5% choline, and 73.0% basic feed). After induction of HF diet for 16 weeks, they were divided into the model group and GXNT group depending on serum lipid levels and body weight and simultaneously continued to be fed HF diet for 12 weeks. Among them, minipigs in the GXNT group were given GXNT at a dose of 162 mg/kg/d (namely, 4 tablets/10 kg/d, equivalent to 3 times the clinical therapeutic dose) for the12 consecutive weeks.

### 2.3. Physical Sign Assessment and Fat Distribution Measurement

After 12 weeks of administration, the minipigs were weighed on an empty stomach. Following anesthesia, the body length, chest circumference, and abdominal circumference were measured to calculate the habitus index with the formula: habitus index = (chest circumference + abdominal circumference)/Body length × 100. Likewise, the retroperitoneal fat, mesenteric fat, greater omentum fat, and fat around the testis were weighed to calculate the total fat weight. Meanwhile, the fat thickness of the front, middle, and rear back was measured.

### 2.4. Blood Lipid Detection

After GXNT treatment for 0, 4, 8, and 12 weeks, pigs fasted for 16 h. Subsequently, the whole blood was collected and centrifuged at 3000 rpm for 15 min. The serum was separated to detect the levels of TC, TG, HDL-C, and LDL-C under the guidance of kit instructions (Medicalsystem Biotechnology Co., Ltd., Ningbo, China). The atherosclerosis index (AI) was calculated with the formula: AI = (TC − HDL‐C)/HDL‐C.

### 2.5. Detection of Serum Inflammation, Oxidative Stress, and Vascular Endothelial Injury-Related Indicators

The commercially available kits of ox-LDL, CRP, TNF-*α*, IL-1*β*, SOD, and MDA were purchased from NanJing JianCheng Bioengineering Institute (Nanjing, China), and vWF and ET-1 were bought from MultiSciences (Lianke) Biotech Co., Ltd. (Hangzhou, China). The corresponding indexes were detected using the ELISA method following the protocol.

### 2.6. Ultrasound Examination of Cardiac Function

Prior to slaughter, the pigs were anesthetized for examination of left ventricular structure and function using an ultrasound instrument (Feiyinuo Technology Co., Ltd., Suzhou, China). We checked the indicators including interventricular septum thickness at end-diastole (IVSd), interventricular septal thickness at end-systole (IVSs), left ventricular end-diastolic diameter (LVIDd), left ventricular end-systolic diameter (LVIDs), left ventricular posterior wall thickness (LVPWd), left ventricular end-systolic posterior wall thickness (LVPWs), ejection fraction (EF), and fraction shortening (FS).

### 2.7. Histopathological Observation of Vascular Tissue

#### 2.7.1. Sudan IV Staining to Observe Lipid Deposition in the Aorta

The whole aorta from the aortic arch to the iliac artery was taken, fixed in 10% formaldehyde for 24 hours, then stained with Sudan IV dye (Sigma, USA) for 15 minutes, rinsed, and photographed. Image-Pro Plus 6.0 software was used to analyze lipid deposition in the thoracic aorta, abdominal aorta, and whole aorta.

#### 2.7.2. HE Staining to Observe the Pathological Morphology of Coronary Artery Tissue

After the animals were sacrificed, the coronary artery was isolated, immediately fixed in 10% formaldehyde, then dehydrated, transparent, embedded in wax, cut into 5 *μ*m slices, patched, stained with HE dye (Thermo, USA), and finally mounted. Pathological sections of vascular tissue were scanned with a 2.0 RS Nana Zoomer digital slide scanner (Hamamatsu, Japan). NDP.view 2 software was used to measure the intima-media thickness (IMT), lumen area (LA), inner elastic lamina area (IELA), and external elastic lamina area (EELA), followed by calculating the neointimal area, media area (MA), and neointimal area/media area (NIA/MA) and neointimal area/internal elastic layer area (NIA/IELA) with the calculation formula: NIA = IELA − LA, MA = EELA − IELA.

#### 2.7.3. Oil Red “O” Staining to Observe Lipid Deposition in Coronary Artery Tissue

Frozen sections of fresh coronary artery tissue with a thickness of 8 *μ*m were fixed in neutral formaldehyde for 15 min, stained with newly prepared and filtered oil red “O” solvent (Sigma, USA) for 15 min, differentiated with 60% isopropanol for seconds, washed with running water, immersed with hematoxylin for 5 min, washed again with water, and finally mounted. Then, pathological sections were scanned with a 2.0 RS Nana Zoomer digital slide scanner (Hamamatsu, Japan), and the lipid deposition was analyzed using Image-Pro Plus 6.0 software.

#### 2.7.4. Observation of the Expression of NF-*κ*B, ICAM-1, and MMP-9 in Coronary Artery Tissue by Immunohistochemistry

Paraffin sections of coronary artery tissue were deparaffinized, repaired by microwave heating, and then cooled in running water, blocked with 3% H_2_O_2_ solution for 10 min, and washed with PBS for 3 min. Then, the sections were incubated with primary anti-NF-*κ*B (Abcam, UK), ICAM-1 (Absin, China), and MMP-9 (Proteintech, USA) at 4°C overnight, respectively. After washing, it was incubated with the secondary antibody for 1 h at room temperature, colored with DAB (ASGB-BIO, China), counterstained with hematoxylin, dehydrated, and mounted. The primary antibody was replaced with PBS as a negative control. Brownish-yellow or light yellow represented the positive substance. The pathological sections were scanned with a 2.0 RS Nana Zoomer digital slide scanner (Hamamatsu, Japan), and the positive expression area and total area in the field of vision were analyzed with Image-Pro Plus 6.0 software. The positive expression rate was calculated with the formula of positive expression area/total area × 100%.

### 2.8. Fecal Metagenomic Analysis for Microbiome and Function

#### 2.8.1. Fecal DNA Extraction and Sequencing

DNA was extracted from fecal samples by the CTAB method as previously described [12]. Briefly, each 200 mg feces were added to the CTAB lysate, incubated at 65°C, reversed to fully dissociate the sample, and centrifuged. Afterward, the supernatant was added with mixtures of phenol (PH = 8.0), chloroform, and isoamyl alcohol (*v*/*v*/*v*, 25 : 24 : 1), inverted, and centrifuged (12000 rpm, 10 min). The supernatant was added with a mixture of chloroform and isoamyl alcohol (*v*/*v*, 24 : 1), inverted thoroughly, and centrifuged (12000 rpm, 10 min). Then, the supernatant was again added with isopropanol, inverted several times, precipitated at -20°C, and centrifuged (12000 rpm, 10 min). After discarding the supernatant, the DNA pellets were washed with 1 mL 75% ethanol twice, air-dried on the benchtop or at room temperature, dissolved with ddH_2_O, and incubated (55-60°C, 10 min) if necessary. In addition, RNA could be removed from the DNA samples using 1 *μ*L RNase A (37°C, 15 min). DNA integrity and purity were separately verified by 1% agarose gel electrophoresis and NanoDrop spectrophotometry (NanoDrop, Germany), and the concentrations were quantified using Qubit Fluorometric Quantification (Thermo Fisher, USA). According to the Illumina protocol of NEBNext Ultra DNA Library Prep Kit, a DNA library was constructed and high-throughput sequencing was conducted on the Illumina NovaSeq platform, following the manufacturer's guidelines.

#### 2.8.2. Sequencing Data Analysis

KneadData software was used for raw data quality control (based on Trimmomatic) and dehosted (based on Bowtie2) prior to acquiring valid sequences (clean data). And FastQC was used to test the rationality and effectiveness of quality control. Microbial databases were downloaded from Kraken2 (http://ccb.jhu.edu/software/kraken2/) to identify the species in the sample, and then, Bracken (http://ccb.jhu.edu/software/bracken/) was used to predict the relative abundance of species. Based on Bracken's absolute abundance and annotation information, *β* diversity analysis and construction of abundance profile were performed on the Tutools platform (https://www.cloudtutu.com). Using HUMAnN2 software (based on DIAMOND), the reads of each sample were mapped to the KEGG database (https://www.kegg.jp/). At last, the annotation information and relative abundance table of each functional database were obtained according to the corresponding relationship between the UniRef90 ID and the database.

### 2.9. Targeted Metabolomics for SCFAs, TMAO, and BAs

#### 2.9.1. Detection of TMAO in Feces and Serum Based on LC-MS

Each 10 mg feces were mixed with 300 *μ*L 1% formic acid-acetonitrile solvent (4°C), ground twice at 50 Hz for 60 s, sonicated 10 min at room temperature, incubated 12 h at 4°C, and then centrifuged at 12,000 rpm for 20 min at 4°C. Subsequently, 20 *μ*L fecal supernatant or serum was accurately pipetted, mixed with 10 *μ*L internal standard and 750 *μ*L 1% formic acid-acetonitrile solvent, vortexed for 30 s, and centrifuged at 12,000 rpm for 5 min at 4°C. The supernatant was filtered with a 0.22 *μ*m filter and transferred to analysis.

TMAO was detected by LC-MS on an ACQUITY UPLC® BEH HILIC column (2.1 mm × 100 mm, 1.7 *μ*m, Waters). The injection volume was 5 *μ*L, and the column temperature was 40°C with acetonitrile as mobile phase A and water as mobile phase B (containing 0.1% formic acid and 10 mM ammonium formate) at a flow rate of 0.4 mL/min. Gradient elution conditions as follows: 0-1 min, 80% A; 1-2 min, 80-70% A; 2-2.5 min, 70% A; 2.5-3 min, 70-50% A; 3-3.5 min, 50% A; 3.5-4 min, 50-80% A; and 4-6 min, 80% A. MS conditions were set to electrospray ionization (ESI) source and ESI+ ionization mode. The ion source temperature was 500°C, the voltage was 5000 V, the collision gas was 6 psi, the curtain gas was 30 psi, and the atomizing gas and auxiliary gas were both 50 psi. Scans were performed using multiple reaction monitoring (MRM).

#### 2.9.2. Detection of Fecal SCFA Based on GC-MS

Each 50 mg sample was prepared, mixed with 50 *μ*L 15% phosphoric acid, 100 *μ*L 125 *μ*g/mL internal standard solution, and 400 *μ*L ether, homogenized for 1 min, and centrifuged at 12000 rpm for 10 min at 4°C.

GC-MS technology detects fecal SCFA profiles based on an Agilent HP-INNOWAX capillary column (30 m∗0.25 mm ID∗0.25 *μ*m) with a split inlet, 1 *μ*L of injection volume, and 10 : 1 split ratio. The inlet temperature was 250°C, the ion source temperature was 230°C, the transfer line temperature was 250°C, and the quadrupole temperature was 150°C. The programmed temperature was initially set to 90°C, heated to 120°C at 10°C/min, then heated to 150°C at 5°C/min, and finally heated to 250°C at 25°C/min for 2 min. The carrier gas was helium at a flow rate of 1.0 mL/min. MS conditions are as follows: electron impact ionization (EI) source, SIM scan mode, and 70 eV electron energy.

#### 2.9.3. Detection of Fecal BAs Based on GC-MS

Each 50 mg sample was accurately weighed, extracted by 1000 *μ*L methanol solvent (-20°C) by vortexing 60 s, vibrated using glass beads (50 Hz, 60 s), repeated the above steps at least 2 times, sonicated for 30 min at room temperature, centrifuged at 4°C (12000 rpm, 10 min), and finally filtered with a 0.22 *μ*m filter.

The contents of BAs in feces were detected by LC-MS using an ACQUITY UPLC® BEH C18 column (2.1 × 100 mm, 1.7 *μ*m, Waters, USA) with 5 *μ*L of injection volume, 40°C of column temperature, 0.01% formic acid water as mobile phase A, and acetonitrile as phase B. Gradient elution conditions were 0-4 min, 25% B; 4-9 min, 25-30% B; 9-14 min, 30-36% B; 14-18 min, 36-38% B; 18-24 min, 38-50% B; 24-32 min, 50-75% B; 32-35 min, 75-100% B; and 35-38 min, 100-25% B. The flow rate was 0.25 mL/min. MS conditions were ESI source and ESI- ionization mode. The ion source temperature was 500°C, the ion source voltage was 4500 V, the collision gas was 6 psi, the curtain gas was 30 psi, and the atomizing gas and auxiliary gas were both 50 psi. Scans were also performed using MRM.

### 2.10. Statistical Analysis

Data were processed using SPSS 24.0 software (SPSS Inc., Chicago, IL, USA). All measurement data were tested for normal distribution, expressed as mean ± standard error ($\bar{x}\pm \text{SEM}$). *T*-test was used for data comparison between two groups, and one-way ANOVA was used to compare data between multiple groups. The statistical graphs were drawn with GraphPad Prism 8.0 software (GraphPad Software Inc., San Diego, CA, USA). All statistics were two-tailed tests, and a *P* value < 0.05 was considered statistically significant.

## 3. Results

### 3.1. Effects of GXNT on General Signs of the CA Model

**Figure 1:**
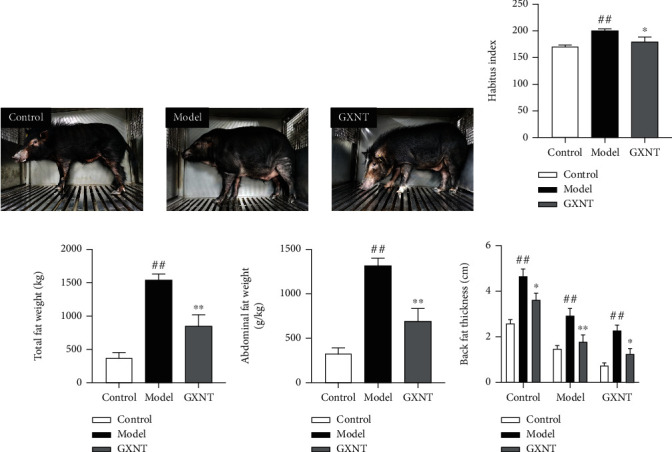
Effects of GXNT on physical signs of the CA model. (a) Representative pictures of habitus. (b) Habitus index. (c) Total fat weight. (d) Abdominal fat weight. (e) Backfat thickness. *n* = 6 in each group. Data are presented as the means ± SE. *^##^P* < 0.01 vs. the control group; ^∗^*P* < 0.05 or ^∗∗^*P* < 0.01 vs. the model group.

During the experiment period, there were no obvious abnormalities of general signs, such as spirits, activity, food intake, feces, and urine of minipigs in the control group. However, after HF diet induction, pigs in the model group exhibited greasy coats, hair loss, obesity, laziness, and lethargy, which were notably improved after GXNT administration for 12 weeks (Figure 1(a)). In addition, habitus index (Figure 1(b)), total fat weight (Figure 1(c)), abdominal fat weight (Figure 1(d)), and backfat thickness (Figure 1(e)) significantly increased in model group (*P* < 0.01). Noticeably, GXNT decreased these indexes (*P* < 0.05, *P* < 0.01).

### 3.2. Effects of GXNT on Blood Lipids of the CA Model

**Figure 2:**
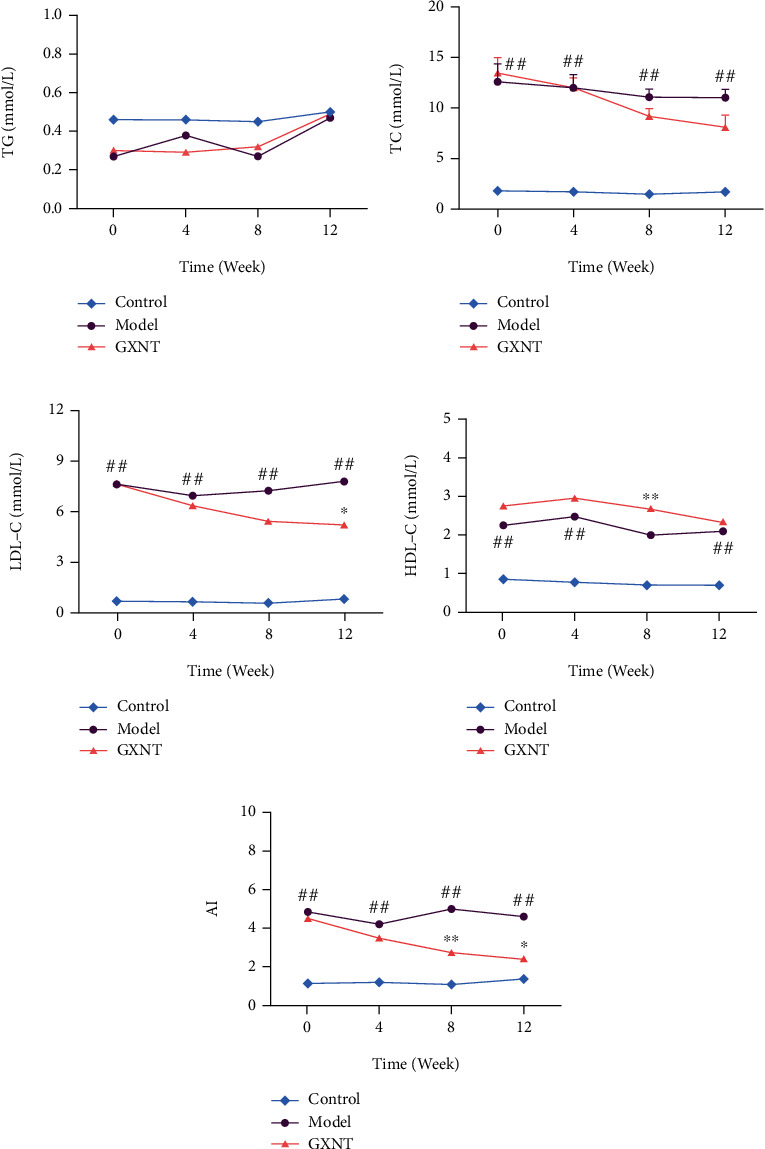
Effects of GXNT on blood lipids of the CA model. Changes of (a) TG, (b) TC, (c) LDL-C, and (d) HDL-C levels and (e) AI during 12 weeks of GXNT administration. *n* = 6 in each group. Data are presented as the means ± SE. ^#^*P* < 0.05 or *^##^P* < 0.01 vs. the control group; ^∗^*P* < 0.05 or ^∗∗^*P* < 0.01 vs. the model group.

The serum level of TG (Figure 2(a)) in the model group significantly decreased at 0 and 8 weeks compared to the control group (*P* < 0.05), whereas TC (Figure 2(b)), LDL-C (Figure 2(c)), and HDL-C (Figure 2(d)) and the AI index (Figure 2(e)) in the model group were much higher than those in the control group (*P* < 0.01). After GXNT administration, the LDL-C level significantly decreased at the 12^th^ week (*P* < 0.05), as well as the AI index at the 8^th^ and 12^th^ weeks (*P* < 0.05, *P* < 0.01). Meanwhile, GXNT could significantly increase HDL-C after 8 weeks of GXNT administration (*P* < 0.01).

### 3.3. Effects of GXNT on Serum Inflammatory Factors of the CA Model

**Figure 3:**
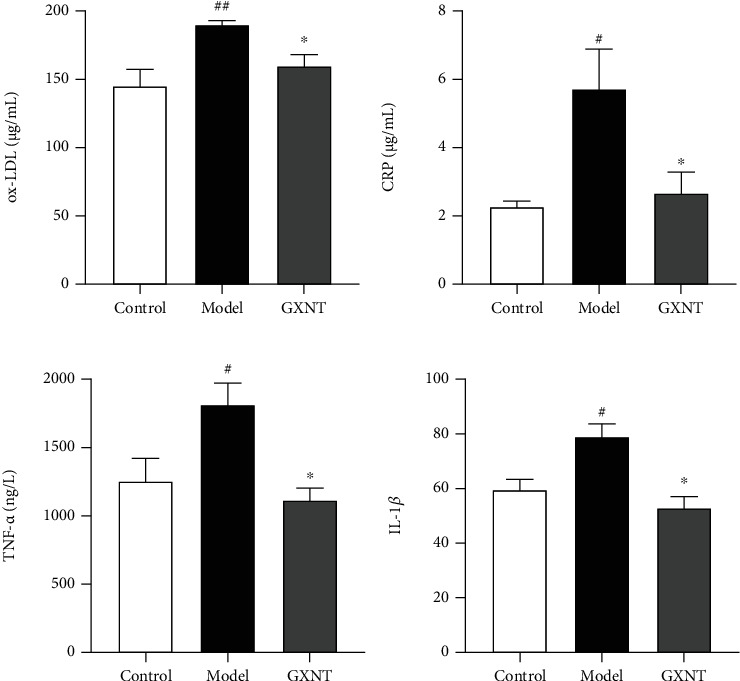
Effects of GXNT on serum inflammatory factors of the CA model. (a) ox-LDL, (b) CRP, (c) TNF-*α*, and (d) IL-1*β* after 12 weeks of GXNT treatment. *n* = 6 in each group. Data are presented as the means ± SE. ^#^*P* < 0.05 or *^##^P* < 0.01 vs. the control group; ^∗^*P* < 0.05 or ^∗∗^*P* < 0.01 vs. the model group.

To evaluate the anti-inflammatory effects of GXNT on the CA model in minipigs induced by HF diet, serum levels of ox-LDL, CRP, TNF-*α*, and IL-1*β* were detected. The serum levels of ox-LDL (Figure 3(a)), CRP (Figure 3(b)), TNF-*α* (Figure 3(c)), and IL-1*β* (Figure 3(d)) in the model group significantly increased compared to the control group (*P* < 0.05, *P* < 0.01), which markedly reduced after GXNT treatment (*P* < 0.05, *P* < 0.01).

### 3.4. Effects of GXNT on Serum SOD and MDA of the CA Model

**Figure 4:**
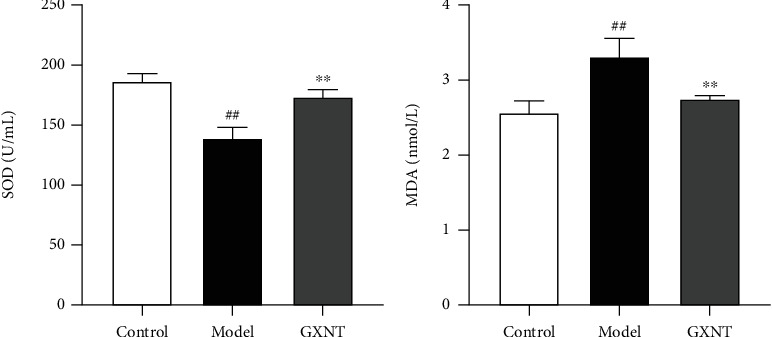
Effects of GXNT on serum SOD and MDA of the CA model. (a) SOD activity. (b) MDA content. *n* = 6 in each group. Data are presented as the means ± SE. *^##^P* < 0.01 vs. the control group; ^∗∗^*P* < 0.01 vs. the model group.

SOD and MDA are important markers of oxidative stress. As shown in Figure 4, we found that the serum SOD activity (Figure 4(a)) in the model group significantly decreased compared to the control group (*P* < 0.01), while the MDA content (Figure 4(b)) significantly increased (*P* < 0.01). Fortunately, GXNT could remarkably ameliorate these changes (*P* < 0.01).

### 3.5. Effects of GXNT on Serum ET-1 and vWF of the CA Model

**Figure 5:**
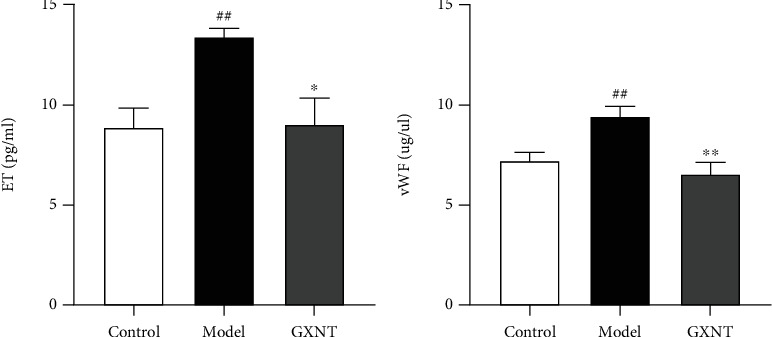
Effects of GXNT on serum ET-1 and vWF of the CA model. The contents of (a) ET-1 and (b) vWF after GXNT treatment for 12 weeks. *n* = 6 in each group. Data are presented as the means ± SE. *^##^P* < 0.01 vs. the control group; ^∗^*P* < 0.05 or ^∗∗^*P* < 0.01 vs. the model group.

Both ET-1 and vWF can reflect endothelial injury. They were measured for observing the protective effect of GXNT on the endothelium. As shown in Figure 5, the serum levels of ET (Figure 5(a)) and vWF (Figure 5(b)) in the model group were much higher than those in the control group (*P* < 0.01). After GXNT treatment, the ET and vWF levels significantly decreased (*P* < 0.05, *P* < 0.01).

### 3.6. Effect of GXNT on Left Ventricular Structure and Function of the CA Model

**Figure 6:**
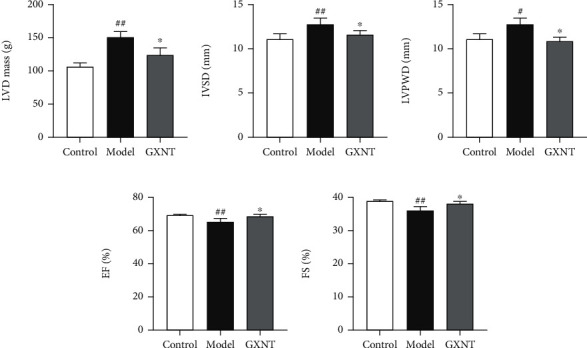
Effects of GXNT on left ventricular structure and function of the CA model induced by HF diet. (a) LVd mass. (b) IVSd. (c) LVPWd. (d) EF. (e) FS. *n* = 6 in each group. Data are presented as the means ± SE. ^#^*P* < 0.05 or *^##^P* < 0.01 vs. the control group; ^∗^*P* < 0.05 vs. the model group.

Patients with CA are often associated with reduced cardiac function; thus, the impact of GXNT on heart function was also evaluated. Compared with the control group, LVd mass (Figure 6(a)), IVSd (Figure 6(b)), and LVPWd (Figure 6(c)) in the model group significantly increased (*P* < 0.05, *P* < 0.01), while EF (Figure 6(d)) and FS (Figure 6(e)) significantly decreased (*P* < 0.01). GXNT treatment could distinctly decrease LVd mass, IVSd, and LVPWd (*P* < 0.05) and increase EF and FS compared with the model group (*P* < 0.05).

### 3.7. Effects of GXNT on Lipid Deposition in Aortic Vessels of the CA Model

**Figure 7:**
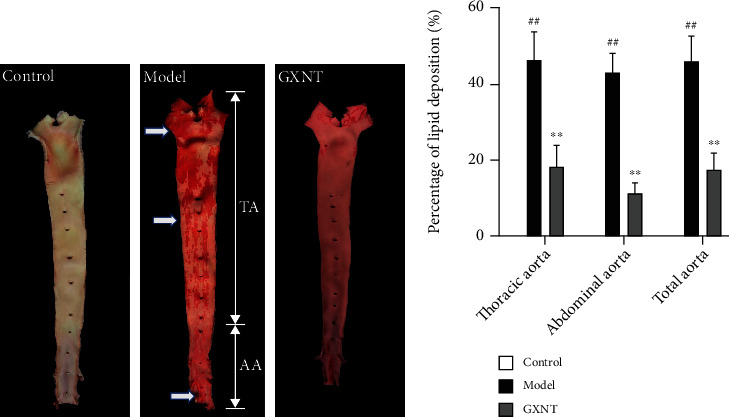
Effects of GXNT on lipid deposition in aortic vessels of the CA model. (a) The representative figures of Sudan IV staining of aortic vessels. (b) Quantitative results of lipid deposition percentage in aortic vessels. *n* = 6 in each group. Data are presented as the means ± SE. *^##^P* < 0.01 vs. the control group; ^∗∗^*P* < 0.01 vs. the model group.

The results of Sudan IV staining showed the red-stained lipid in the thoracic aorta and abdominal aorta in the model group (indicated by white arrows) was much more than that in the control group, which was greatly improved in the GXNT group (Figure 7(a)). Subsequently, quantitative analysis (Figure 7(b)) further showed that the lipid deposition in the thoracic aorta, abdominal aorta, and whole aorta in the model group markedly increased compared to the control group (*P* < 0.01). And GXNT administration could significantly reduce the lipid deposition (*P* < 0.01).

### 3.8. Effects of GXNT on the Pathological Morphology of Coronary Artery Tissue of the CA Model

**Figure 8:**
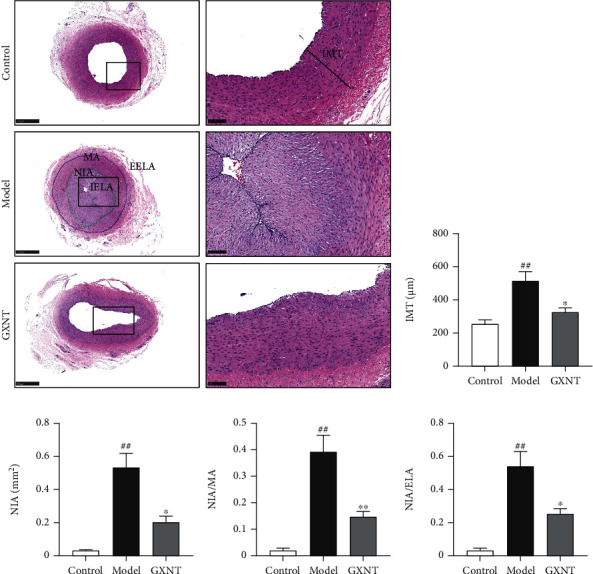
Effects of GXNT on the pathological morphology of coronary artery tissue of the CA Model. (a) Representative images of HE staining of the coronary vessels. Left: lower magnification (scale bars, 500 *μ*m); right: higher magnification (scale bars, 100 *μ*m) of the black-boxed area. (b) IMT. (c) NIA. (d) NIA/MA. (e) NIA/IELA. IMT: intima-media thickness; NIA: neointimal area; NIA/MA: neointimal area/media area; NIA/IELA: neointimal area/internal elastic layer area. *n* = 6 in each group. Data are presented as the means ± SE. *^##^P* < 0.01 vs. the control group; ^∗^*P* < 0.05 or ^∗∗^*P* < 0.01 vs. the model group.

In the control group, the intima and media structures of coronary artery tissue were intact and clear, without obvious abnormalities. In the model group, the intima of the coronary artery had obvious hyperplasia, infiltration of inflammatory cells such as macrophages, increased foam cells, severe atherosclerotic plaques, fibrous cap formation, and serious vascular stenosis, which were attenuated by GXNT as shown in Figure 8(a). Similarly, quantitative analysis showed that IMT (Figure 8(b)), NIA (Figure 8(c)), NIA/MA (Figure 8(d)), and NIA/IELA (Figure 8(e)) in the model group significantly increased as compared to the control group (*P* < 0.01), which could be clearly decreased by GXNT treatment (*P* < 0.05, *P* < 0.01).

### 3.9. Effects of GXNT on Lipid Deposition of Coronary Artery Tissue of the CA Model

**Figure 9:**
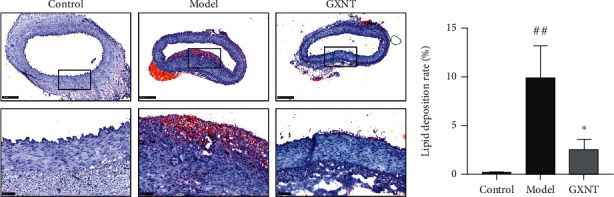
Effects of GXNT on lipid deposition of coronary artery tissue of the CA model. (a) Representative images of oil red “O” staining of coronary vessels. Top: lower magnification (scale bars, 500 *μ*m); bottom: higher magnification (scale bars, 100 *μ*m) of the black-boxed area. (b) Quantitative analysis of coronary vascular lipid deposition. *n* = 6 in each group. Data are presented as the means ± SE. *^##^P* < 0.01 vs. the control group; ^∗^*P* < 0.05 vs. the model group.

The results of oil red “O” staining showed that only a small amount of lipid was deposited in coronary artery tissue in the control group, while extensive lipid deposition appeared in the model group, and the lipid was visually reduced in the GXNT group, as shown in Figure 9(a). Compared with the control group, the lipid deposition rate of the coronary artery in the model group significantly increased using quantitative assessment (*P* < 0.01) (Figure 9(b)). On the contrary, the lipid deposition rate was significantly decreased after GXNT administration (*P* < 0.05).

### 3.10. Effects of GXNT on the Protein Expressions of NF-*κ*B, TNF-ɑ, and MMP-9 in Coronary Artery Tissue of the CA Model

**Figure 10:**
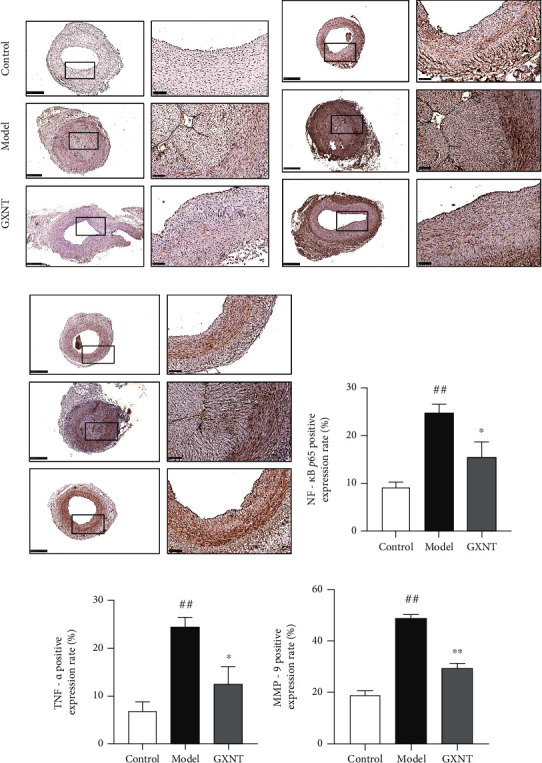
Effects of GXNT on the expressions of NF-*κ*B, TNF-ɑ, and MMP-9 in coronary artery tissue of the CA model. The protein expressions of (a) NF-*κ*B, (b) TNF-ɑ, and (c) MMP-9 by immunohistochemical staining. Left: lower magnification (scale bars, 500 *μ*m); right: higher magnification (scale bars, 100 *μ*m) of the black-boxed area. The positive expression rates of (d) NF-*κ*B, (e) TNF-ɑ, and (f) MMP-9 by quantitative analysis. *n* = 6 in each group. Data are presented as the means ± SE. *^##^P* < 0.01 vs. the control group; ^∗^*P* < 0.05 or ^∗∗^*P* < 0.01 vs. the model group.

The immunohistochemical staining results of NF-*κ*B, TNF-*α*, and MMP-9 proteins in coronary vessels were shown in Figures 10(a)–10(c), separately. Compared with the control group, the apparent positive expressions of NF-*κ*B, TNF-ɑ, and MMP-9 proteins in the intima and middle layers in the model group could be observed, which had a visual reduction in GXNT group. Furthermore, quantitative analysis showed that the positive expression rates of NF-*κ*B (Figure 10(d)), TNF-ɑ (Figure 10(e)), and MMP-9 (Figure 10(f)) in the model group significantly increased as compared to the control group (*P* < 0.01). And the positive expression rates in the GXNT group were much less than those in the model group (*P* < 0.05, *P* < 0.01).

### 3.11. Effects of GXNT on the Composition of Gut Microbiota in the CA Model

**Figure 11:**
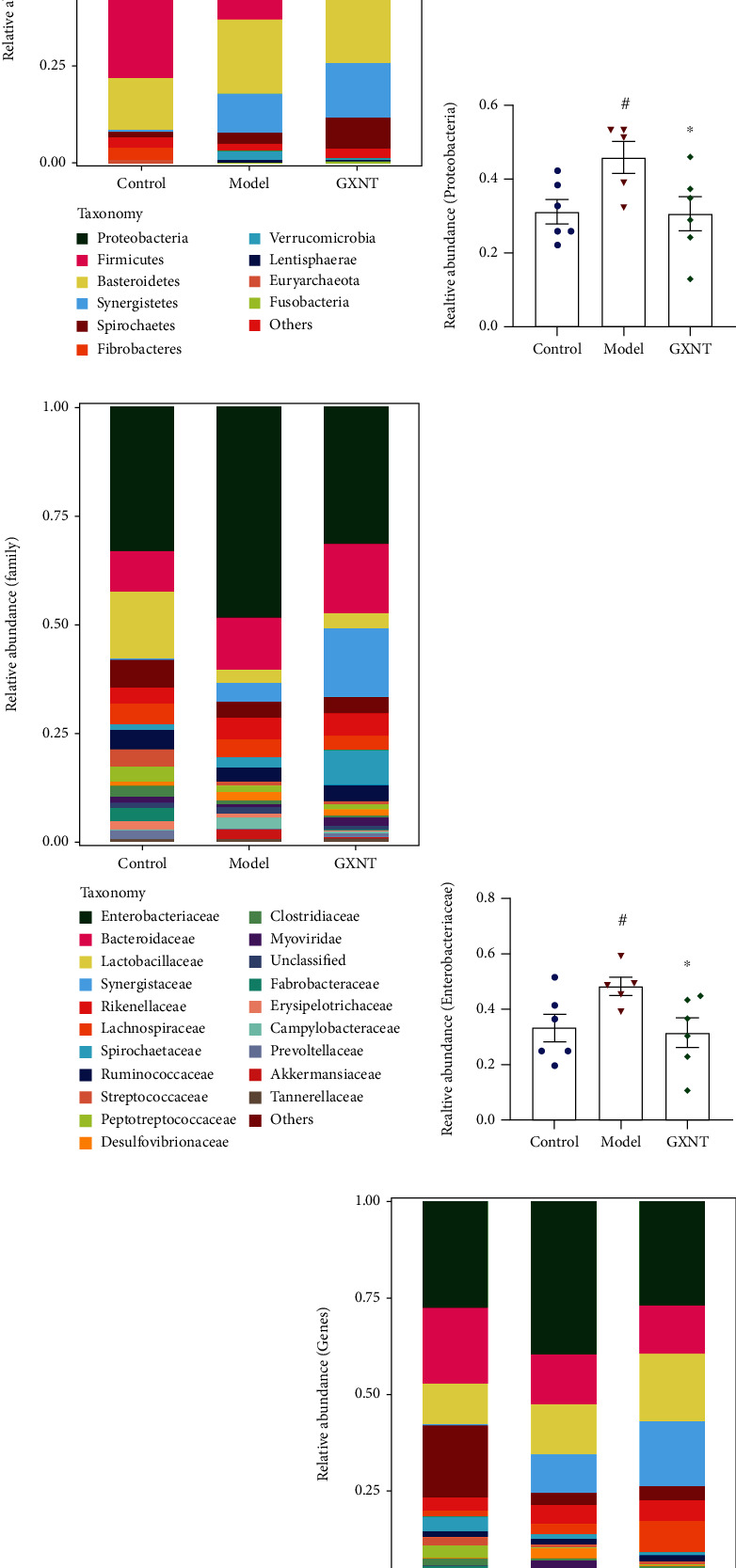
Effects of GXNT on the composition of gut microbiota in the CA model. (a) Effect of GXNT on the diversity of gut microbiota in the CA Model. (b, d, g) Effects of GXNT on the relative abundance distribution of gut microbiota at the level of phylum (top10), family (top20), and genus (top20) in the CA model. (c, e, f, h, i) Effects of GXNT on the relative abundance of *Proteobacteria*, *Enterobacteriaceae*, *Prevotellaceae*, *Escherichia*, and *Prevotella*. *n* = 6 in both the control and GXNT groups, *n* = 5 in the model group. Data are presented as the means ± SE. ^#^*P* < 0.05 vs. the control group; ^∗^*P* < 0.05 vs. the model group.

To observe the *β* diversity changes, we carried out methods of principal component analysis (PCA) based on the similarity coefficient matrix and principal coordinates analysis (PCoA) based on the Bray-Curtis distance matrix. Significant differences existed in the composition and structure between the control group and model group, which could be improved by GXNT, as shown in Figure 11(a).

The relative abundance of the gut microbiota was analyzed, and it was found that there were significant differences at the phylum, family, and genus levels. The top 10 species in relative abundance at the phylum level were selected to construct the abundance profile as shown in Figure 11(b). *Proteobacteria*, *Bacteroidetes*, and *Firmicutes* were the three dominant phyla in the control group, model group, and GXNT group. The relative abundance of *Proteobacteria* in the model group significantly increased as compared with the control group (*P* < 0.05) (Figure 11(c)). And GXNT administration could decrease its relative abundance (*P* < 0.05).

As to the levels of family and genus, the top 20 species were selected to construct abundance profiles (Figures 11(d) and 11(g), respectively). *Enterobacteriaceae* was the dominant bacteria shared by 3 groups. Compared with the control group, the relative abundance of *Enterobacteriaceae* in the model group significantly increased (*P* < 0.05) (Figure 11(e)), while the abundance of *Prevotellaceae* obviously decreased (*P* < 0.05) (Figure 11(f)). GXNT treatment could distinctly regulate their relative abundance (*P* < 0.05). At the level of the genus, *Escherichia*, belonging to the *Enterobacteriaceae* family, was the common dominant bacteria in the 3 groups. The relative abundance of *Escherichia* in the model group significantly increased as compared with the control group (*P* < 0.05) (Figure 11(h)), and the relative abundance of *Prevotella* decreased (*P* < 0.05) (Figure 11(i)). After GXNT administration, the relative abundance of *Escherichia* apparently reduced (*P* < 0.05), and the relative abundance of *Prevotella* significantly increased (*P* < 0.05).

### 3.12. The Effects of GXNT on Gut Microbiota Function Based on KEGG Analysis

The KEGG orthology database is the basic database for studying gene function. Using the KO database, genes that have the same function can be aggregated. Then, differences in metabolic pathways (Pathways) and functional modules (Modules) between groups can be analyzed based on the KO.

As for KO, the gut microbiota genes associated with the functions of bile acid metabolism (K03455), choline metabolism (K00108), carnitine metabolism (K22443 and K22444), acetate kinase production (K00925), and lipopolysaccharide biosynthesis (K00748) in the model group significantly rose as compared to the control group (*P* < 0.05, *P* < 0.01), while those related to short-chain fatty acid transport (K02106), butyrate kinase production (K00929), and propionate kinase production (K00932) clearly decreased (*P* < 0.05, *P* < 0.01). All of these genes involved in the functions above were significantly regulated by GXNT (*P* < 0.05), as shown in Table 1.

**Figure 12:**
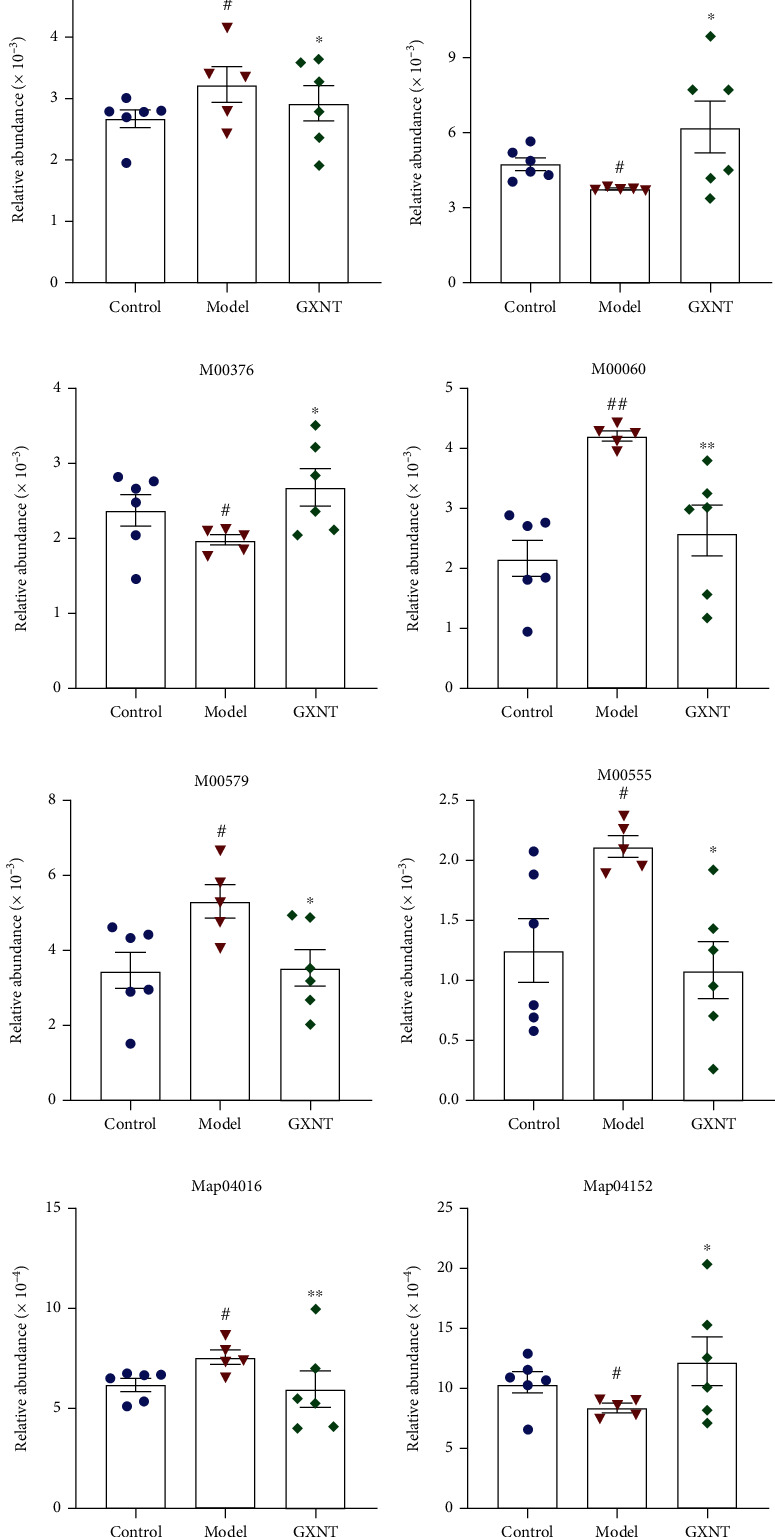
Effects of GXNT on the module function and pathway of gut microbiota in the CA model. Effects of GXNT on the relative abundance of (a) Entner-Doudoroff pathway-related microbiota, (b) pentose phosphate cycle-related microbiota, (c) hydroxypropionate pathway-related microbiota, (d) lipopolysaccharide production-related microbiota, (e) phosphoacetyltransferase-acetate kinase pathway-related microbiota, (f) choline production-related microbiota, (g) MAPK pathway-related microbiota, and (h) AMPK pathway-related microbiota. M00008: Entner-Doudoroff pathway, glucose-6P=>glyceraldehyde-3P+pyruvate. M00167: reductive pentose phosphate cycle, glyceraldehyde-3P=>ribulose-5P. M00376: 3-hydroxypropionate bi-cycle. M00060: lipopolysaccharide biosynthesis, KDO2-lipid A. M00579: phosphate acetyltransferase-acetate kinase pathway, acetyl-CoA=>acetate. M00555: betaine biosynthesis, choline=>betaine. Map04016: MAPK signaling pathway-plant. Map04152: AMPK signaling pathway. *n* = 6 in both the control and GXNT groups, *n* = 5 in the model group. ^#^*P* < 0.05 or ^##^*P* < 0.01 vs. the control group; ^∗^*P* < 0.05 or ^∗∗^*P* < 0.01 vs. the model group.

In terms of functional modules, the relative abundance of microbiota linked to the Entner-Doudoroff pathway (Figure 12(a)), lipopolysaccharide production (Figure 12(d)), phosphate acetyltransferase-acetate kinase pathway (Figure 12(e)), and choline production (Figure 12(f)) significantly rose compared to the control group (*P* < 0.05, *P* < 0.01), and the relative abundance of microbiota in connection with the pentose phosphate cycle (Figure 12(b)) and hydroxypropionic acid cycle (Figure 12(c)) significantly reduced (*P* < 0.05). GXNT treatment could evidently modulate the relative abundance of the function-related bacteria mentioned above (*P* < 0.05, *P* < 0.01). As to metabolic pathways, compared with the control group, the bacterial genes related to the MAPK signaling pathway (Figure 12(g)) significantly increased in model group (*P* < 0.05), and those related to the AMPK signaling pathway (Figure 12(h)) significantly decreased (*P* < 0.05). GXNT treatment could regulate the corresponding abundance of bacteria related to the above signaling pathways (*P* < 0.05, *P* < 0.01).

### 3.13. Effects of GXNT on Metabolites of CA-Related Gut Microbiota

#### 3.13.1. Effects of GXNT on Metabolite SCFAs in the CA Model

**Figure 13:**
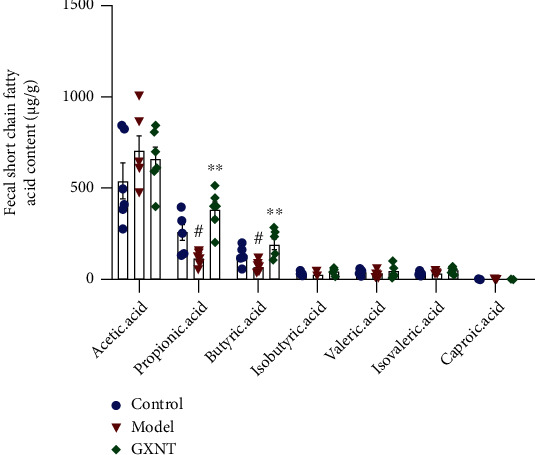
Effects of GXNT on fecal SCFAs in the CA model. *n* = 6 in each group. ^#^*P* < 0.05 vs. the control group; ^∗∗^*P* < 0.01 vs. the model group.

The contents of fecal SCFAs, including acetic acid, propionic acid, butyric acid, isobutyric acid, valeric acid, isovaleric acid, and caproic acid, were detected by the GC-MS method. The results in Figure 13 showed that acetic acid, propionic acid, and butyric acid were the main SCFAs, accounting for about 90%-95% of the total content. The propionic acid and butyric acid contents in the model group significantly decreased as compared with the control group (*P* < 0.05), which could be significantly increased by GXNT (*P* < 0.01).

#### 3.13.2. Effects of GXNT on TMA-Related Metabolites in the CA Model

**Figure 14:**
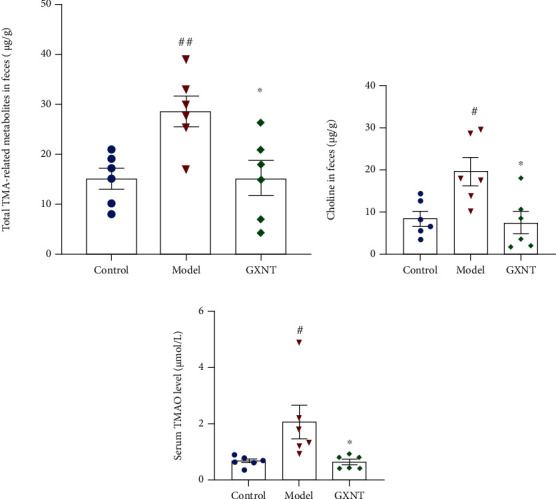
Effects of GXNT on TMA-related metabolites in the CA model. Effects of GXNT on (a) fecal total TMA-related metabolites, (b) fecal choline, and (c) serum TMAO. *n* = 6 in each group. ^#^*P* < 0.05 or ^##^*P* < 0.01 vs. the control group; ^∗^*P* < 0.05 vs. the model group.

The contents of fecal TMA-related metabolites and serum TMAO, including choline, betaine, TMAO, creatinine, and carnitine, were detected by the LC-MS method, as shown in Figure 14. According to the results, we found that fecal total TMA-related metabolites in the model group significantly increased as compared to the control group (*P* < 0.01; Figure 14(a)), as well as fecal choline content (*P* < 0.05; Figure 14(b)) and serum TMAO (*P* < 0.05; Figure 14(c)). After GXNT treatment, all of them were significantly decreased (*P* < 0.05).

#### 3.13.3. Effects of GXNT on Metabolite BAs in the CA Model

**Figure 15:**
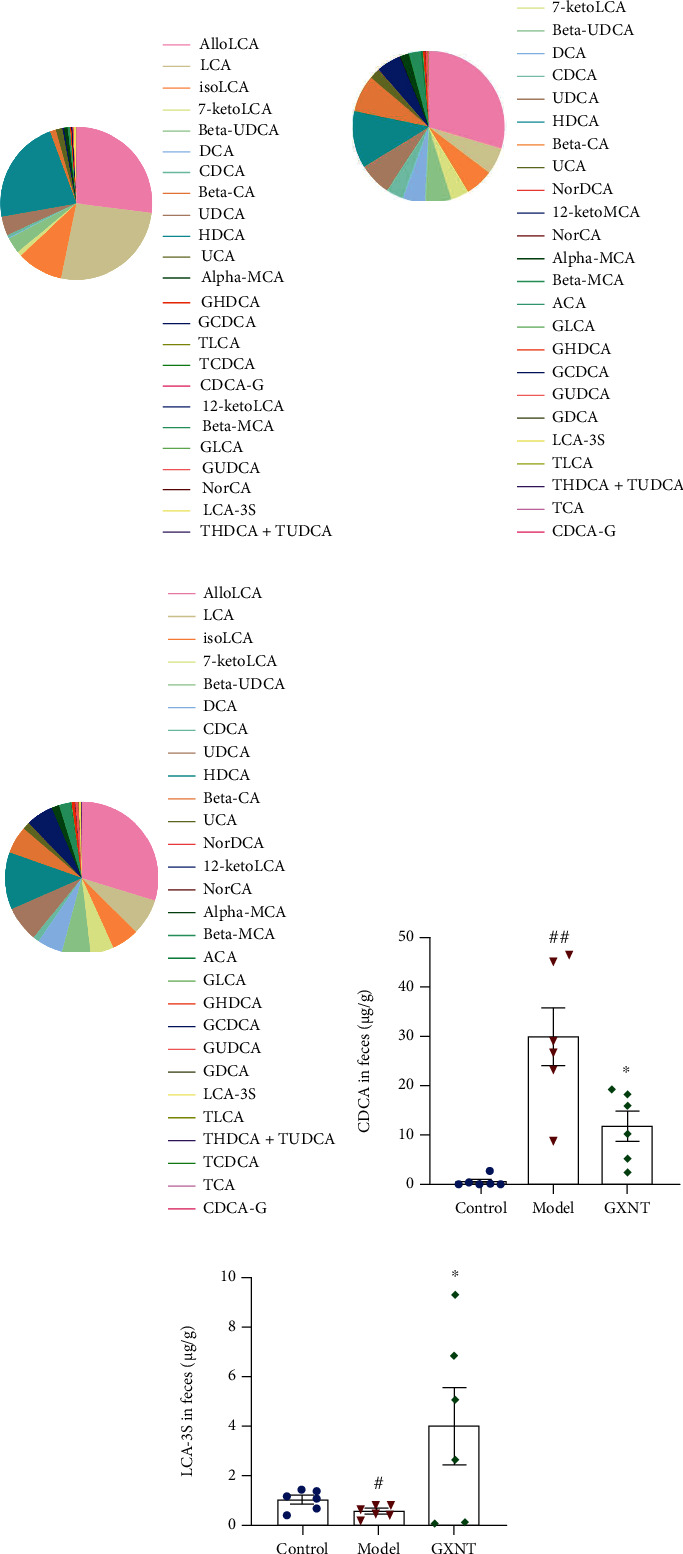
Effects of GXNT on fecal BAs in the CA model. The contents of metabolite BAs in the (a) control group, (b) model group, and (c) GXNT group were detected using LC-MS. Effects of GXNT on fecal (d) CDCA and (e) LCA-3S. *n* = 6 in each group. ^#^*P* < 0.05 or ^##^*P* < 0.01 vs. the control group; ^∗^*P* < 0.05 vs. the model group.

The contents of fecal metabolite BAs, such as lithocholic acid (LCA), allolithocholic acid (alloLCA), chenodeoxycholic acid (CDCA), cholic acid (CA), deoxycholic acid (DCA), glycinechenodeoxycholic acid (GCDCA), glycocholic acid (GCA), taurocholic acid (TCA), and lithocholic acid 3-sulfate sodium salt (LCA-3S), were detected using the LC-MS method. The effects of GXNT on fecal BAs in 3 groups were shown (Figures 15(a)–15(c)). Among the fecal BA metabolites, the CDCA (Figure 15(d)) in the model group significantly increased (*P* < 0.01) compared to the control group, whereas LCA-3S (Figure 15(e)) significantly decreased (*P* < 0.05). GXNT treatment could clearly reduce CDCA (*P* < 0.05) and distinctly increased LCA-3S (*P* < 0.05).

## 4. Discussion

GXNT is clinically used for treating CHD and ischemic cardiomyopathy with significant curative effects [13]. Our previous pharmacological experiments confirmed that GXNT has the functions of reducing whole blood viscosity, improving blood rheology abnormalities, antimyocardial ischemia, antithrombosis, and vasodilation [14–19]. In this study, we established a CA model induced by a HF diet in Tibetan minipigs and simultaneously interfered with GXNT. It was found that GXNT could regulate blood lipids, inhibit platelet aggregation, improve cardiac dysfunction, reduce vascular lipid deposition, inhibit vascular inflammation, protect vascular endothelium, and reduce AS lesions. Using metagenomic and targeted metabolomics analysis, we revealed that GXNT could regulate the composition of gut microbiota and related metabolites in the CA model, thereby preventing AS formation.

Dyslipidemia is an independent risk factor for AS [20]. The relationship between diet, lipid level, and AS is well established, and atherosclerotic lesions in animals and humans appear to be associated with elevated cholesterol and excess fat intake [21]. Dyslipidemia can damage vascular endothelial cells and consequently stimulate vascular intimal hyperplasia. Endometrial injury is an initiating factor for AS, with injury markers ET-1 and vWF increasing [22]. vWF can activate platelets to release coagulation substances for participating in platelet aggregation, thrombosis, and AS plaque formation, thereby resulting in coronary stenosis, insufficient blood supply, and decreased cardiac function. As an important factor, oxidative stress can also damage vascular endothelial cells. The level of MDA is one of the indicators reflecting the degree of oxidative stress, and the activity of SOD reflects the ability to scavenge reactive oxygen species. Our results showed that GXNT can regulate dyslipidemia, reduce visceral and arterial lipid deposition, reduce AS plaques, resist oxidation and endothelial damage, inhibit platelet aggregation, and improve cardiac function, suggesting that GXNT can resist AS plaque formation and protect endothelial function and cardiac function, accordingly.

AS is a chronic low-grade inflammatory process [23, 24]. In the early stage of AS, LDL infiltrates and is oxidized into ox-LDL after endothelial damage, followed by inflammatory factor expression, the adherence of monocytes to endothelial cells, foam cell forming by phagocytosis of ox-LDL. With the progression of AS, inflammatory factors increase, such as TNF-ɑ, IL-1*β*, and IL-6, which aggravate the development of AS plaques. As an independent risk factor for AS, CRP directly participates in the formation and aggregation of AS. The study showed that GXNT could inhibit MDA content, enhance SOD activity, and reduce the levels of proinflammatory factors, thus contributing to restraining the development of AS.

NF-*κ*B is an important transcription factor in the process of an inflammatory response, which can regulate the expression of various inflammatory genes related to the occurrence of AS [25]. It is reported that TNF-ɑ can activate NF-*κ*B, initiate the transcription and expression of various inflammatory factors, and lead to the occurrence of AS. MMP-9 is considered a biochemical marker of plaque instability, and it can degrade almost all extracellular substances, leading to increased plaque vulnerability. The results of our study showed that GXNT can significantly reduce the expressions of NF-*κ*B, TNF-ɑ, and MMP-9 in AS plaques, indicating that GXNT can reduce the inflammatory response, playing an important role in plaque stability.

Accumulating evidence suggests that gut microbiota play a critical role in cardiovascular disease. There were significant differences in the composition and diversity of gut microbiota in patients with different types of CHD, and *Proteobacteria* increased in CA patients [26], which is in line with our results. Another research reported that the relative abundances of *Escherichia-Shigella* and *Enterococcus* in patients with coronary artery disease significantly increased, while the relative abundances of *Faecalibacterium* and *Roseburia* significantly decreased, which may be related to functions such as lipopolysaccharide synthesis and propionic acid metabolism enhancement [27]. The current study showed decreased gut microbiota diversity in the CA model induced by a HF diet in minipigs, which is consistent with our previous findings [11], and GXNT can improve the bacterial composition to a certain extent. GXNT could decrease the relative abundances of *Proteobacteria*, *Enterobacteriaceae*, and *Escherichia* and increase the relative abundances of *Prevotellaceae* and *Prevotella*. *Proteobacteria* are known as microbial hallmarks of gut dysbiosis and are associated with immune inflammation and metabolic dysfunction. After GXNT intervention, the relative abundance of *Proteobacteria* was significantly reduced, speculating that *proteobacteria* were the characteristic bacteria of GXNT in treating AS. Jie et al. found that the abundance of *Enterobacteriaceae family* and *Streptococcus species* increased in patients with coronary artery disease, while the abundance of *Roseburia Intestinalis* and *Faecalibacterium prausnitzii* decreased, which was associated with reduced fermentative capacity and enhanced proinflammatory properties [28]. Studies have shown that *Prevotella* are the most abundant bacteria in the human gut and play a crucial role in nutrition and metabolism, such as degrading dietary fiber, fermenting polysaccharides, lowering cholesterol [29]. Thus, GXNT may ameliorate the formation of a HF diet-induced CA by modulating gut microbiota composition.

In addition, functional changes in gut microbiota were predicted using metagenomics, and targeted metabolites were detected based on the predicted results. The results showed that GXNT could affect the relative abundances of bacteria related to bile acid metabolism, choline biosynthesis, SCFA production in a HF diet-induced CA model, and TMAO, SCFAs, and BAs are closely related to AS [30]. TMAO has been recognized as a biomarker for predicting AS [31]. TMA is produced by TMA-forming microbial communities (e.g., *Escherichia coli*) from a HF diet mainly containing choline and carnitine. Subsequently, it is further oxidized to TMAO by hepatic flavin monooxygenase, resulting in AS occurrence through multiple mechanisms [32]. TMAO can induce the activation of MAPK and NF-*κ*B in endothelial cells and smooth muscle cells and promote the expression of downstream inflammatory factors such as IL-8 and IL-1*β*. High levels of TMAO can also affect lipid metabolism, increasing the risk of cardiovascular disease by reducing reverse cholesterol transport and altering bile acid transport, composition, pool size, etc. [33]. In this experiment, GXNT can reduce the generation of TMA metabolites and lower the circulating TMAO level, thereby preventing the AS formation, which may be related to the regulation of *Escherichia*. SCFAs are the main products of intestinal dietary fiber fermentation. Acetic acid, propionic acid, and butyric acid are the most abundant SCFAs, accounting for about 95% of the total SCFAs. SCFAs can regulate energy metabolism, and blood pressure, fight inflammation and improve insulin resistance by binding to corresponding receptors [34]. Particularly, propionate and butyrate can inhibit the NF-*κ*B signaling pathway by regulating receptor signaling, reduce the production of downstream inflammatory factors, and prevent AS progression. Butyrate can enhance intestinal barrier function by regulating the expression of claudin, which may be achieved by activating AMPK or downregulating the expression of claudin. In this study, GXNT may increase intestinal acetate and propionate content, possibly affecting energy metabolism, intestinal permeability, and inflammatory factor production. Additionally, BAs are the main metabolites of cholesterol. They are now considered signaling molecules that interact with cell membranes and nuclear receptors and have regulatory effects on physiological processes such as energy balance and glycolipid metabolism. In the colorectal, primary bile acids, such as CA and CDCA, are metabolized to secondary bile acids, including DCA, LCA, and UDCA [35]. Conjugated or unconjugated bile acids are reabsorbed through the enterohepatic circulation, while LCA and UDCA are mostly excreted in the feces. Amazingly, gut microbes can regulate the ratio of bile acids. When the body is in an unhealthy state, gut microbes can cause a decrease in secondary bile acids, an increase in primary bile acids, activation of FXR, and downregulation of bile acid production, leading to cholesterol increase and AS occurrence [36]. Our results showed that GXNT can clearly reduce CDCA and increase LCA-3S, hinting that GXNT can promote primary bile acid conversion and increase bile acid excretion, thereby reducing cholesterol level. Overall, GXNT may also exert anti-AS function by influencing the metabolites of the gut microbiota.

The therapeutic effect of GXNT on AS may be associated with its phenolic acids. In previous studies, we have identified 14 active components using LC-MS [37], and 7 phenolic acids of which were quantified, including tanshinol, protocatechualdehyde, chlorogenic acid, caffeic acid, ferulic acid, rosmarinic acid, and salvianolic acid B. The phenolic acids have been widely reported for AS treatment. Tanshinol has a pronounced anti-inflammatory effect involving TLR2 and macrophages through the NF-*κ*B signaling pathway, which supports the novel application of DSS in the treatment of relevant diseases including AS and ischemic or ischemic/perfusion injury of the heart and brain [38]. Chlorogenic acid mitigates ox-LDL-induced endothelial oxidative stress and mitochondrial dysfunction by activating SIRT1 and modulating the AMPK/PGC-1 signaling pathway [39]. The mix of chlorogenic acid and caffeic acid exhibits a high antioxidant effect, which can reduce lipid storage in macrophages by a reduction in the expression of transcription factors C/EBP*β* and PPAR-*γ*1 [40]. Gu et al. reported that FA could significantly ameliorate atherosclerotic injury, which may be partly by modulating gut microbiota and lipid metabolism via the AMPK*α*/SREBP1/ACC1 pathway [41]. Rosmarinic acid inhibits endothelial dysfunction which is shown in diabetic atherosclerosis through downregulating the p38-FOXO1-TXNIP pathway and inhibiting inflammasome activation [42]. Salvianolic acid B can attenuate the development of AS, the anti-AS effect of which is related to regulating the YAP/TAZ/JNK signaling pathway [43]. Moreover, these phenolic acids are linked to gut health. Substances containing phenolic acids, such as protocatechuic acid, chlorogenic acid, caffeic acid, ferulic acid, and rosmarinic acid, have the function of suppressing intestinal inflammation and modulating gut microbial populations [44, 45]. Thus, we speculate that the phenolic acids contained in GXNT can regulate the composition and structure of the gut microbiota in the AS model, as well as the production of metabolites, exerting anti-inflammatory and anti-AS effects. Meanwhile, the phenolic acids can also be metabolized by the gut microbiota to generate beneficial components, which are absorbed by the body and contribute to AS treatment. In general, the therapeutic effect of GXNT on AS may be the result of the interaction between phenolic acids and gut microbiota, which needs further study.

## 5. Conclusions

GXNT has the functions of regulating blood lipids, antioxidative stress, antivascular endothelial injury, inhibiting platelet aggregation, anti-inflammatory, and reducing AS plaque area, thereby exerting an anti-CA effect. The mechanism may be closely related to regulating the composition of the gut microbiota and bacterial metabolites such as TMAO, SCFAs, and BAs. We found that GXNT could modulate the relative abundance of *Proteobacteria*, *Enterobacteriaceae*, *Escherichia*, *Prevotellaceae*, and *Prevotella*, possibly involved in AS development, which may be the target bacteria for GXNT in AS therapy. The experiment provides an experimental basis for the clinical application of GXNT in the treatment of CA and new inspiration for researching the underlying mechanism. However, the composition of GXNT is complex, and its specific mechanism of action needs to be further studied.

## Figures and Tables

**Table 1:** Effects of GXNT on the relative abundance of functional genes of the gut microbiota (means ± SE).

KO entries	Relative abundance			Gene name	Definition
	Control	Model	GXNT		
K03455 (×10^−5^)	2.22 ± 0.50	6.58 ± 1.22^#^	3.53 ± 0.69^∗^	TC.BASS	Bile acid : Na+ symporter, BASS family
K00108 (×10^−4^)	1.53 ± 0.25	2.66 ± 0.16^##^	1.61 ± 0.36^∗^	betA,CHDH	Choline dehydrogenase
K22443 (×10^−4^)	0.80 ± 0.20	1.48 ± 0.10^#^	0.90 ± 0.20^∗^	cntA	Carnitine monooxygenase subunit
K22444 (×10^−4^)	0.48 ± 0.12	0.91 ± 0.09^#^	0.49 ± 0.14^∗^	cntB	Carnitine monooxygenase subunit
K02106 (×10^−5^)	2.44 ± 0.26	1.24 ± 0.11^##^	3.07 ± 0.58^∗^	atoE	Short-chain fatty acids transporter
K00925 (×10^−4^)	7.11 ± 0.38	10.69 ± 1.01^#^	7.22 ± 0.80^∗^	ackA	Acetate kinase
K00929 (×10^−5^)	12.72 ± 1.90	5.64 ± 0.84^#^	15.96 ± 2.93^∗^	buk	Butyrate kinase
K00932 (×10^−4^)	2.73 ± 0.48	0.86 ± 0.07^#^	1.80 ± 0.33^∗^	tdcD	Propionate kinase
K00748 (×10^−4^)	1.38 ± 0.23	2.39 ± 0.33^#^	1.23 ± 0.30^∗^	lpxB	Lipid-A-disaccharide synthase

Note: *n* = 6 in both the control and GXNT groups, *n* = 5 in the model group. ^#^*P* < 0.05 or ^##^*P* < 0.01 vs. the control group; ^∗^*P* < 0.05.

## Data Availability

All related data have presented in the manuscript.

## Supplementary Materials

Supplementary Materials Supplementary Table 1: identification results of main compounds in GXNT.
