# Supplementary material for: Guanxinning Tablet Attenuates Coronary Atherosclerosis via Regulating the Gut Microbiota and Their Metabolites in Tibetan Minipigs Induced by a High-Fat Diet
Source: J Immunol Res. 2022 Jul 28;2022:7128230. doi: 10.1155/2022/7128230 (PMC9352486; doi:10.1155/2022/7128230)
Supplement: Supplementary Materials — Supplementary Table 1: identification results of main compounds in GXNT. [file 7128230.f1.pdf]

Supplementary Table 1: Identification results of main compounds in GXNT

Main ions in the positive mode	Main ions in the negative mode	Molecular weight	Compound name	Content in extract (%)
166[M+H]+；149[M+H-NH3]+；120[M+H-HCOOH]+；103[M+H-HCOOH-NH3]+	164[M-H]−；147[M-H-NH3]−	165	Phenylalanine	-
199[M+H]+	197[M-H]-；179[M-H-H2O]-；133[M-H-H2O-HCOOH]-	198	Tanshinol	0.580
205[M+H]+；187[M+H-H2O]+	n.d.b	204	Senkyunolide B	-
n.d.	137[M-H]-；109[M-H-CO]-	138	Protocatechualdehyde	0.135
355[M+H]+、163[M+H-192]+	707[2M-H]-；353[M-H]-；191[M-H-162]-	354	Chlorogenic acid	0.084
181[M+H]+；163[M+H-H2O]+	179[M-H]-；135[M-H-CO2]-	180	Caffeic acid	0.097
195[M+H]+；177[M+H-H2O]+；149[M+H-H2O-CO]+；117[M+H-H2O-CO-CH3OH]+	193[M-H]-；178[M-H-CH3·]-•；134[M-H-CH3·-CO2]-•	194	Ferulic acid	0.362
419[M+H]+；177[M+H-CO2-198]+	835[2M-H]-；417[M-H]-；373[M-H-CO2]-；175[M-H-CO2-198]-	418	Salvianolic acid D	-

225[M+H]+；207[M+H-H2O]+；189[M+H-H2O-H2O]+；161[M+H-H2O-HCOOH]+	n.d.	224	Senkyunolide I	-
361[M+H]+；181[M+H-180]+；163[M+H-198]+	359[M-H]-；197[M-H-162]-；179[M-H-180]-；161[M-H-198]-	360	Rosmarinic acid	0.551
495[M+H]+；297[M+H-198]+	493[M-H]-；295[M-H-198]-	494	Isosalvianolic acid A	-
719[M+H]+；521[M+H-198]+；323[M+H-198-198]+	717[M-H]-；519[M-H-198]-；321[M-H-198-198]-	718	Salvianolic acid B	4.676
495[M+H]+；297[M+H-198]+	987[2M-H]-；493[M-H]-；295[M-H-198]-	494	Salvianolic acid A	-
n.d.	491[M-H]-；293[M-H-198]-	492	Isosalvianolic acid C	-

Note: n.d. (not detected) means not detected, no or below the detection limit.
